# Cisplatin induces tolerogenic dendritic cells in response to TLR agonists via the abundant production of IL-10, thereby promoting Th2- and Tr1-biased T-cell immunity

**DOI:** 10.18632/oncotarget.9260

**Published:** 2016-05-09

**Authors:** Woo Sik Kim, Hongmin Kim, Kee Woong Kwon, Sin-Hyeog Im, Bo Ryeong Lee, Sang-Jun Ha, Sung Jae Shin

**Affiliations:** ^1^ Department of Microbiology, Institute for Immunology and Immunological Diseases, Brain Korea 21 PLUS Project for Medical Science, Yonsei University College of Medicine, Seoul, South Korea; ^2^ Academy of Immunology and Microbiology (AIM), Institute for Basic Science (IBS), Pohang, South Korea; ^3^ Division of Integrative Biosciences and Biotechnology (IBB), Pohang University of Science and Technology (POSTECH), Pohang, South Korea; ^4^ Department of Biochemistry, College of Life Science & Biotechnology, Yonsei University, Seoul, South Korea

**Keywords:** cisplatin, tolerogenic dendritic cells, toll-like receptor, IL-10, Tr1 polarization, Immunology and Microbiology Section, Immune response, Immunity

## Abstract

Although many advantageous roles of cisplatin (*cis*-diamminedichloroplatinum (II), CDDP) have been reported in cancer therapy, the immunomodulatory roles of cisplatin in the phenotypic and functional alterations of dendritic cells (DCs) are poorly understood. Here, we investigated the effect of cisplatin on the functionality of DCs and the changes in signaling pathways activated upon toll-like receptor (TLR) stimulation. Cisplatin-treated DCs down-regulated the expression of cell surface molecules (CD80, CD86, MHC class I and II) and up-regulated endocytic capacity in a dose-dependent manner. Upon stimulation with various TLR agonists, cisplatin-treated DCs showed markedly increased IL-10 production through activation of the p38 MAPK and NF-κB signaling pathways without altering the levels of TNF-α and IL-12p70, indicating the cisplatin-mediated induction of tolerogenic DCs. This effect was dependent on the production of IL-10 from DCs, as neither DCs isolated from IL-10^−/−^ mice nor IL-10-neutralized DCs generated tolerogenic DCs. Interestingly, DCs that were co-treated with cisplatin and lipopolysaccharide (LPS) exhibited a decreased immunostimulatory capacity for inducing the proliferation of Th1- and Th17-type T cells; instead, these DCs contributed to Th2-type T cell immunity. Furthermore, *in vitro* and *in vivo* investigations revealed a unique T cell population, IL-10-producing CD3^+^CD4^+^LAG-3^+^CD49b^+^CD25^−^Foxp3^−^ Tr1 cells, that was significantly increased without altering the Foxp3^+^ regulatory T cell population. Taken together, our results suggest that cisplatin induces immune-suppressive tolerogenic DCs in TLR agonist-induced inflammatory conditions via abundant IL-10 production, thereby skewing Th cell differentiation towards Th2 and Tr1 cells. This relationship may provide cancer cells with an opportunity to evade the immune system.

## INTRODUCTION

Cisplatin (*cis*-DDP, *cis*-diamminedichloroplatinum (II)), a platinum-based anti-tumor drug, is one of the most commonly used metal-containing compounds in medicine [1–3]. Cisplatin and its analogues (*i.e.,* carboplatin and oxaliplatin) are among the most potent chemotherapy drugs used for cancer treatment [1, 3]. The discovery of cisplatin as an anti-cancer drug in the 1960s by Rosenberg and colleagues ushered in a new paradigm in cancer treatment [3, 4]. Cisplatin is thought to damage rapidly growing tumor cells *via* the induction of apoptosis following the inhibition of DNA synthesis and repair, resulting in cell cycle arrest at the G1, S, or G2-M phase [1, 5, 6]. Cisplatin has clinical benefits for several types of solid tumors. However, cisplatin treatment is frequently accompanied by toxic side effects and tumor resistance, which in turn leads to secondary malignancies [1–3].

In recent years, medical research has focused on elucidating the mechanisms underlying cancer drugs. The development of new techniques to identify perturbations in cellular functions has increased knowledge of the molecular, physiological and pathological mechanisms of cancer drugs. In particular, emerging evidence has revealed the complex interplay that exists between the host immune system and many anti-cancer drugs. However, little information is available regarding how cisplatin interacts with immune cells. Thus, a better understanding of the molecular mechanisms through which cisplatin induces and suppresses immunological responses is needed to develop and optimize new therapeutic strategies using cisplatin. In particular, cisplatin has been shown to induce immunosuppressive effects through the inhibition of T cell activity [7, 8]. However, little is known about how cisplatin suppresses innate and adaptive immunity.

Immunological interventions for tumor therapy have focused on two aspects: 1) immune cell-based tumor therapy such as dendritic cell (DC)-based tumor immunotherapy, and 2) immune checkpoint inhibition such as blocking PD-1/PD-L1. Although these two approaches differ, both enhance tumor-targeted Th1-type T cell immunity by harnessing immunological power or by overcoming tolerance and suppression [9–12]. In this regard, DCs are the most potent cell type involved in both strategies. In fact, DCs are the most important cell population for activating anti-tumor T cell responses. However, tumors can also directly or indirectly induce DCs to both functionally and phenotypically favor the tumor environment [12–14]. DC activation leads to a cascade of pro- or anti-inflammatory cytokine production, migration to secondary lymphoid tissues, and priming of naïve T cells. Therefore, these cells regulate immune homeostasis and the balance between tolerance and immunity [12, 13]. Most importantly, DCs play a critical role in regulating CD4 and CD8 T cell immunity by controlling Th1, Th2, and Th17 commitment; generating inducible Tregs; and mediating tolerance or immunostimulation [12, 13, 15]. It is believed that distinct DC subsets have evolved to control these different immune outcomes. However, how these DC subsets mount different responses to inflammatory and/or tolerogenic signals to accomplish their divergent functions remains unclear.

The effects of anti-cancer drugs on the immune system remain controversial. However, select chemotherapeutic agents primarily suppress DCs, and the effect of chemotherapeutic drugs on DC function requires further investigation in various inflammatory settings. In this context, we characterized the effect of cisplatin on the function of DCs, which play crucial roles in bridging innate and adaptive immunity. This study describes for the first time the key mechanisms involved in the switch to a tolerogenic DC phenotype that is induced by cisplatin following toll-like receptor (TLR) agonist activation of inflammation and the resulting consequences on T cell polarization.

## RESULTS

### Determination of a cisplatin concentration that does not reduce DC viability

Cisplatin at concentrations ≥ 25 μM or ≥10 μg/ml induces cell death of cancer cell lines and primary cultured cells, such as macrophages, *via* DNA fragmentation [16, 17]. Prior to conducting the current study, the viability of bone marrow-derived dendritic cells (BMDCs) exposed to cisplatin was investigated to determine a cisplatin concentration that does not cause cell death and could therefore be used in subsequent experiments. As expected, a cisplatin concentration over 10 μg/ml showed a cytotoxic effect on BMDCs when measured by MTT assay (Supplementary Figure S1A). Therefore, cisplatin concentrations ≤ 10 μg/ml were used for subsequent experiments, as these concentrations did not reduce cell viability. In addition, no significant decrease in DC viability following co-treatment with 10 μg/ml of cisplatin and 100 ng/ml of lipopolysaccharide (LPS) was observed by MTT assay (Supplementary Figure S1B) or Annexin V and propidium iodide (PI) staining (Supplementary Figure S1C). This finding suggests that cisplatin is not cytotoxic to DCs when used at concentrations below 10 μg/ml. Therefore, concentrations of 1 and/or 5 μg/ml of cisplatin were used for further investigation.

### Cisplatin impairs the phenotypic maturation of LPS-activated DCs

**Figure 1:**
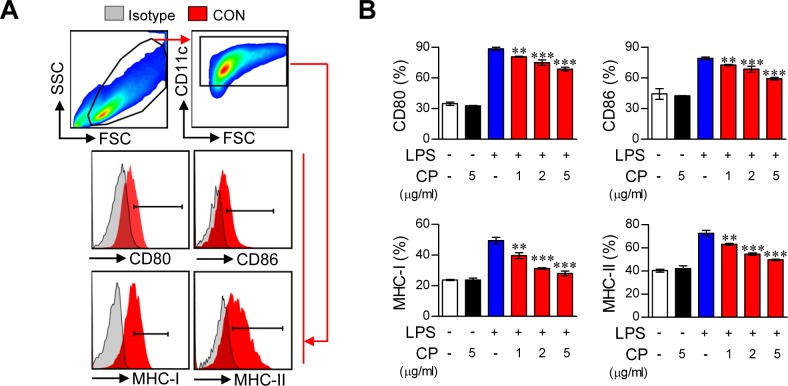
Cisplatin impaired the phenotypic maturation of LPS-activated DCs by down-regulating the expression of CD80, CD86, MHC-I and II in a dose-dependent manner **A.,B.** CD11c^+^ immature BMDCs were cultured for 18 h in the presence of LPS (100 ng/ml), cisplatin (CP, 5 μg/mL), or LPS (100 ng/mL) with cisplatin (1, 2, and 5 μg/mL). The DCs were then stained with anti-CD80, anti-CD86, anti-MHC class I, or anti-MHC class II Abs. **A.** The surface marker expression of each DC set was compared to that of the controls. Isotype control (filled gray histogram), CON (non-stimulated DC, filled red histogram). **B.** The bar graphs show the mean ± SD (*n* = 3 samples) of the percentage of each surface molecule expressed by CD11c^+^ cells. The results are representative of three independent experiments. **p < 0.01 or ***p < 0.001 compared to DCs treated with LPS alone.

Elevated expression of the co-stimulatory molecules CD80 and CD86 as well as MHC class I and II following TLR agonist stimulation is a key feature of mature DCs [18]. However, the immunological actions of cisplatin, especially on DC maturation and function, remain unknown. We therefore investigated whether treatment with cisplatin resulted in phenotypic alteration in DCs upon stimulation with LPS. To accomplish this, CD11c^+^ DCs were identified using the gating strategy shown in Figure 1A. The DCs were cultured for 8 days in OptiMEM supplemented with GM-CSF under standard conditions and then treated with LPS (100 ng/ml), cisplatin (5 μg/mL), or LPS (100 ng/mL) and cisplatin (1, 2, and 5 μg/mL) for one day. The cisplatin treatment significantly decreased the LPS-induced expression of the co-stimulatory molecules CD80 and CD86 and MHC class I and II in a dose-dependent manner, suggesting that cisplatin can alter the DC maturation phenotype. However, the DCs that were treated with cisplatin alone did not show any increases or decreases in these markers (Figure 1B). Importantly, the DCs that were co-treated with cisplatin (5 μg/mL) and LPS (100 ng/mL) showed significant reductions in the levels of the above surface molecules in a time-dependent manner (Supplementary Figure S2A).

### Cisplatin functionally induces TLR agonist-activated DCs toward a tolerogenic phenotype

**Figure 2:**
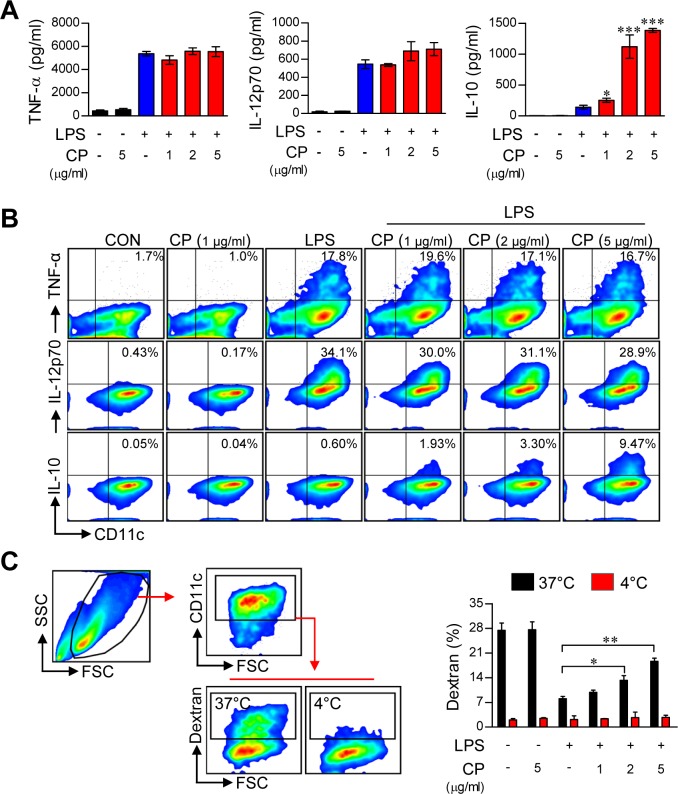
Cisplatin increased IL-10 production and endocytosis in LPS-activated DCs **A.** BMDCs were treated with LPS (100 ng/mL) in the absence or presence of cisplatin at various concentrations (1, 2 and 5 μg/mL) for 18 h. The amounts of TNF-α, IL-12p70, and IL-10 in the culture supernatants were measured by ELISA. The data are shown as the mean ± SD (*n* = 3 samples). One representative plot out of three independent experiments is shown. *p < 0.05 and ***p < 0.001 compared to treatment with LPS alone. **B.** Intracellular TNF-α, IL-12p70 and IL-10 levels in CD11c^+^ DCs. One representative plot from three independent experiments is shown. **C.** The cells were incubated with FITC-dextran either at 37°C for 30 min or at 4°C as a control. Endocytic capacity was determined by assessing the endocytosis of FITC-dextran by flow cytometry (left panel). The bar graphs (right panel) indicate the mean ± SD (*n* = 3 samples) of the percentage of dextran-FITC-positive CD11c^+^ cells. The results are representative of three independent experiments. The values shown represent the mean ± SD (*n* = 3 samples). **p < 0.01 or ***p < 0.001 compared to DCs treated with LPS alone.

The above findings suggest that cisplatin is involved in phenotypic alteration during TLR agonist-induced DC maturation. Thus, we hypothesized that cisplatin influences DC function by regulating cytokine production. To validate this hypothesis, we analyzed cytokine production in cisplatin-treated DCs following stimulation with various TLR ligands, including Pam3-CSK (TLR2), polyI:C (TLR3), LPS (TLR4), IMQ (TLR7), and ODN (TLR9). Surprisingly, for all of the tested TLR agonists, cisplatin dramatically increased the levels of the anti-inflammatory cytokine IL-10 in activated DCs. Furthermore, none of the tested TLR agonists inhibited or increased the production of other pro-inflammatory cytokines, such as TNF-α and IL-12 (Figure 2A and Supplementary Figure S3). These results indicate that cisplatin conditions DCs to produce a high amount of IL-10, independent of the specific TLR pathway being activated. Next, we confirmed that cisplatin strongly induced intracellular IL-10 production in LPS-activated DCs. We found that DCs co-treated with cisplatin and LPS exhibited a significantly higher percentage of IL-10-positive cells and that this percentage increased in a dose-dependent manner, whereas no changes were observed in the numbers of TNF-α-positive or IL-12p70-positive cells (Figure 2B). Additionally, the co-treated DCs showed increased production of IL-10 in a time-dependent manner, whereas no changes were observed in the production of TNF-α or IL-12p70 (Supplementary Figure S2B).

### Cisplatin increases the endocytic activity of LPS-activated DCs

A decrease in endocytosis is a hallmark of mature DCs, whereas tolerogenic DCs maintain higher endocytic ability [19]. To evaluate whether the antigen uptake ability of LPS-activated DCs is increased or decreased by cisplatin treatment, DCs were activated by LPS in the absence or presence of cisplatin and then assessed for their ability to uptake antigens by endocytosis using FITC-conjugated dextran. A significantly lower percentage of FITC-positive DCs were observed among the LPS-stimulated DCs, while the cisplatin and LPS co-treatment enhanced the endocytic activity of DCs in a dose-dependent manner (Figure 2C). These results, together with the reduction of mature DC surface markers and the remarkable enhancement in IL-10 production, strongly suggest that cisplatin endows DCs with a tolerogenic property upon TLR stimulation.

### Cisplatin induces IL-10 secretion through activation of the p38 MAPK and NF-κB signaling pathways

**Figure 3:**
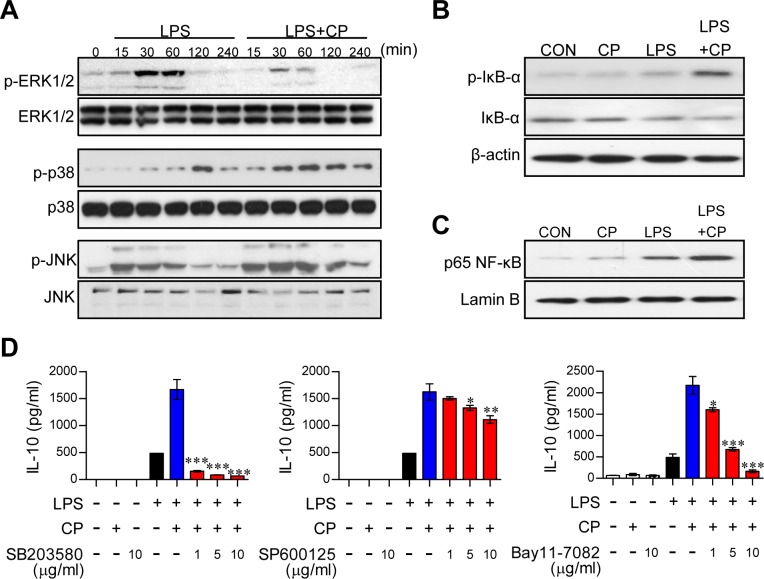
Cisplatin-induced IL-10 production in LPS-activated DCs involved the activation of the p38 MAPK and NF-κB signaling pathways **A.** BMDCs were treated with LPS (100 ng/ml) in the absence or presence of cisplatin (5 μg/ml) for 0, 15, 30, 60, 120, and 240 min. Cell lysates were subjected to SDS-PAGE, and immunoblot analyses were performed using Abs specific to phospho-ERK1/2, phospho-JNK1/2 and phospho-p38. **B.**, **C.** Cell lysates and nuclear lysates were subjected to SDS-PAGE, and immunoblot analyses were performed using Abs specific to phospho-IκB-α (p-IκB-α), non-phospho-IκB-α, and p65 NF-κB for 60 min. β-actin and lamin B were used as loading controls for the cytosolic and nuclear fractions, respectively. One representative plot out of three independent experiments is shown. **D.** BMDCs were treated with pharmacological inhibitors of p38 (SB203580), JNK (SP600125), or NF-κB (Bay11-7082) or DMSO (vehicle control) for 1 h prior to treatment with LPS in the absence or presence of cisplatin for 18 h. IL-10 levels in the culture medium were measured by ELISA. The values shown represent the mean ± SD from one representative plot out of three independent experiments; *p < 0.05, **p < 0.01, or ***p < 0.001 compared to DCs treated with LPS and cisplatin.

Activation of the MAPK, cyclooxegenase-2 (COX-2)/prostaglandin E2 (PGE2) and NF-κB signaling cascades increases IL-10 secretion in DCs [20, 21]. To examine whether these pathways are involved in increased IL-10 production following treatment with cisplatin, the expression and phosphorylation levels of MAPKs, including p38, ERK, and JNK; the phosphorylation and degradation of IκB-α; and the nuclear translocation of NF-κB p65 were investigated by Western blot analysis (Figure 3). In DCs co-treated with cisplatin and LPS, rapid and prolonged p38 phosphorylation and slightly activated JNK phosphorylation was observed compared to DCs treated with LPS alone. Furthermore, ERK1/2 phosphorylation was decreased in the co-treated DCs compared to the DCs treated with LPS alone (Figure 3A). Importantly, treatment with cisplatin alone had no effect on MAPK expression and phosphorylation (Supplementary Figure S4A). Additionally, COX-2 activation and PGE-2 expression were not changed by cisplatin treatment (Supplementary Figure S4B). Cisplatin induced the phosphorylation and degradation of IkB-α and the nuclear translocation of p65 from the cytosol (Figure 3B and 3C). To confirm the involvement of p38, JNK, and NF-κB in the above phenotypes, we next treated cells with specific pharmacological inhibitors (SB203580 for p38, SP600125 for JNK, and Bay11-7082 for NF-κB) and cisplatin and then evaluated IL-10 production following the cisplatin treatment. These pharmacological inhibitors, particularly the p38 inhibitor, significantly abrogated cisplatin-induced IL-10 production in LPS-activated DCs (Figure 3D), suggesting that the p38 and NF-kB signaling cascades are the main pathways responsible for the cisplatin-induced production of IL-10 in LPS-activated DCs.

### Cisplatin treatment conditions DCs to become tolerogenic through the IL-10 cascade

**Figure 4:**
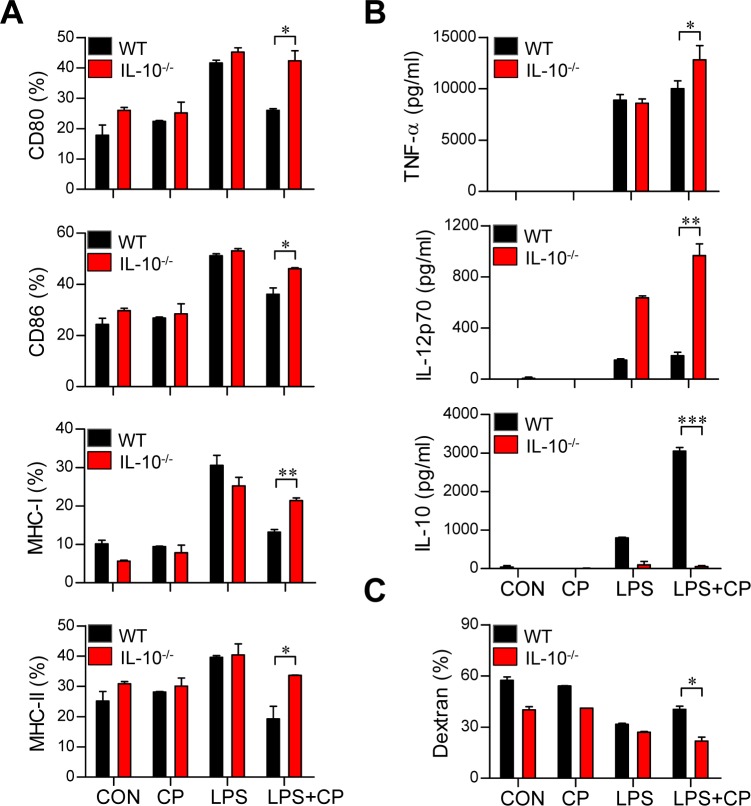
The IL-10 cascade induced cisplatin/LPS-primed DCs to adopt a tolerogenic phenotype **A.**, **B.**, **C.** BMDCs were generated from WT and IL-10^−/−^ mice. The DCs were treated with LPS (100 ng/ml), cisplatin (5 μg/mL), or LPS with cisplatin. **A.** After 18 h, the expression of CD80, CD86, and MHC class I and II on the DCs was assessed. **B.** TNF-α, IL-12p70 and IL-10 levels in the supernatants of DCs from WT and IL-10^−/−^ mice treated with LPS, cisplatin or LPS with cisplatin were measured by ELISA.**C.** Endocytic activity of DCs from WT and IL-10^−/−^ mice treated with LPS, cisplatin or LPS and cisplatin was assessed at 37°C by evaluating the uptake of FITC-conjugated dextran. The results show are representative of three independent experiments and expressed as the mean ± SD (*n* = 3 samples). *p < 0.05, **p < 0.01 and ***p < 0.001 when comparing the WT DCs and IL-10^−/−^ DCs before and after treatment with LPS and cisplatin.

**Figure 5:**
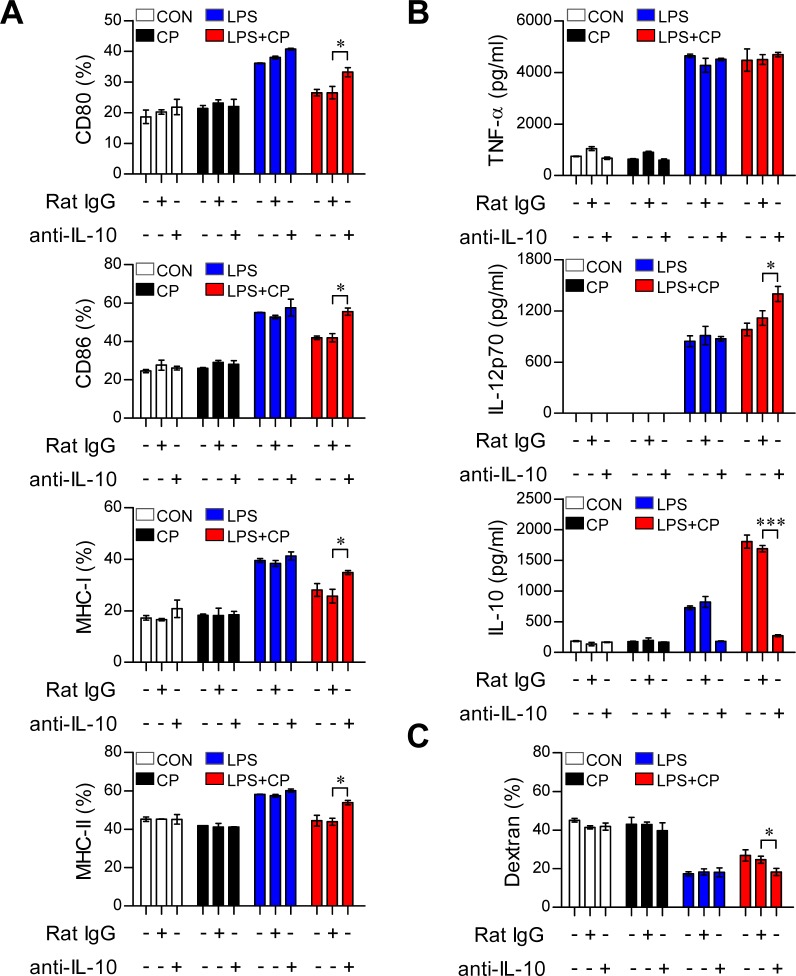
IL-10 neutralization restored the phenotypic and functional maturation of LPS-activated DCs from cisplatin-induced tolerogenic DCs **A.**, **B.**, **C.** BMDCs were treated with a neutralizing anti-IL-10 mAb or rat IgG (isotype control) in the absence or presence of LPS (100 ng/ml) or cisplatin (5 μg/ml). **A.** After 18 h, TNF-α, IL-12 p70 and IL-10 levels in the culture supernatants were measured by ELISA. **B.** The cells were harvested, and the surface molecule expression patterns of CD11c^+^ cells were measured by flow cytometry. **C.** Endocytic capacity was determined by assessing the endocytosis of FITC-dextran after IL-10 neutralization. The percentages of dextran-FITC positive CD11c^+^ cells are indicated. All bar graphs show the mean ± SD (*n* = 3 samples) of one representative plot from three independent experiments. *p < 0.05 and ***p < 0.001 between the anti-IL-10 and IgG isotype control in DCs treated with LPS and cisplatin.

IL-10 induces the maturation and phenotypic alteration of DCs towards a tolerogenic state [22, 23]. Thus, we hypothesized that if cisplatin-induced increases in IL-10 production cause LPS-activated DCs to adopt a tolerogenic phenotype, the absence of IL-10 should restore an activated DC phenotype even in the presence of cisplatin. To investigate whether the IL-10 increase stimulated by cisplatin induces DC phenotypic and functional changes, DCs isolated from IL-10^−/−^ mice were treated with cisplatin, LPS or a combination of cisplatin and LPS (Figure 4). The IL-10^−/−^ DCs displayed the LPS-activated DC phenotype, including elevated levels of surface molecules (Figure 4A) and reduced endocytic activity (Figure 4C). However, increased levels of pro-inflammatory cytokines (TNF-α and IL-12p70) and decreased levels of the anti-inflammatory cytokine IL-10 were observed in the IL-10^−/−^ DCs compared to WT DCs following co-treatment with LPS and cisplatin (Figure 4B). We further confirmed that IL-10 induces LPS-activated DCs to become tolerogenic following cisplatin treatment through experiments in which we blocked the IL-10 cascade with an anti-IL-10 monoclonal antibody (mAb) (Figure 5). Incubation of the co-treated DCs with an anti-IL-10 mAb, but not with control rat IgG, for 18 h restored their surface molecule expression levels (Figure 5A) and IL-12p70 production (Figure 5B) and decreased their endocytic activity (Figure 5C). Collectively, these results indicate that the cisplatin-induced tolerogenic DC phenotype requires IL-10.

### Cisplatin-treated DCs reduce the proliferation of T cells and alter the polarization of T cells

**Figure 6:**
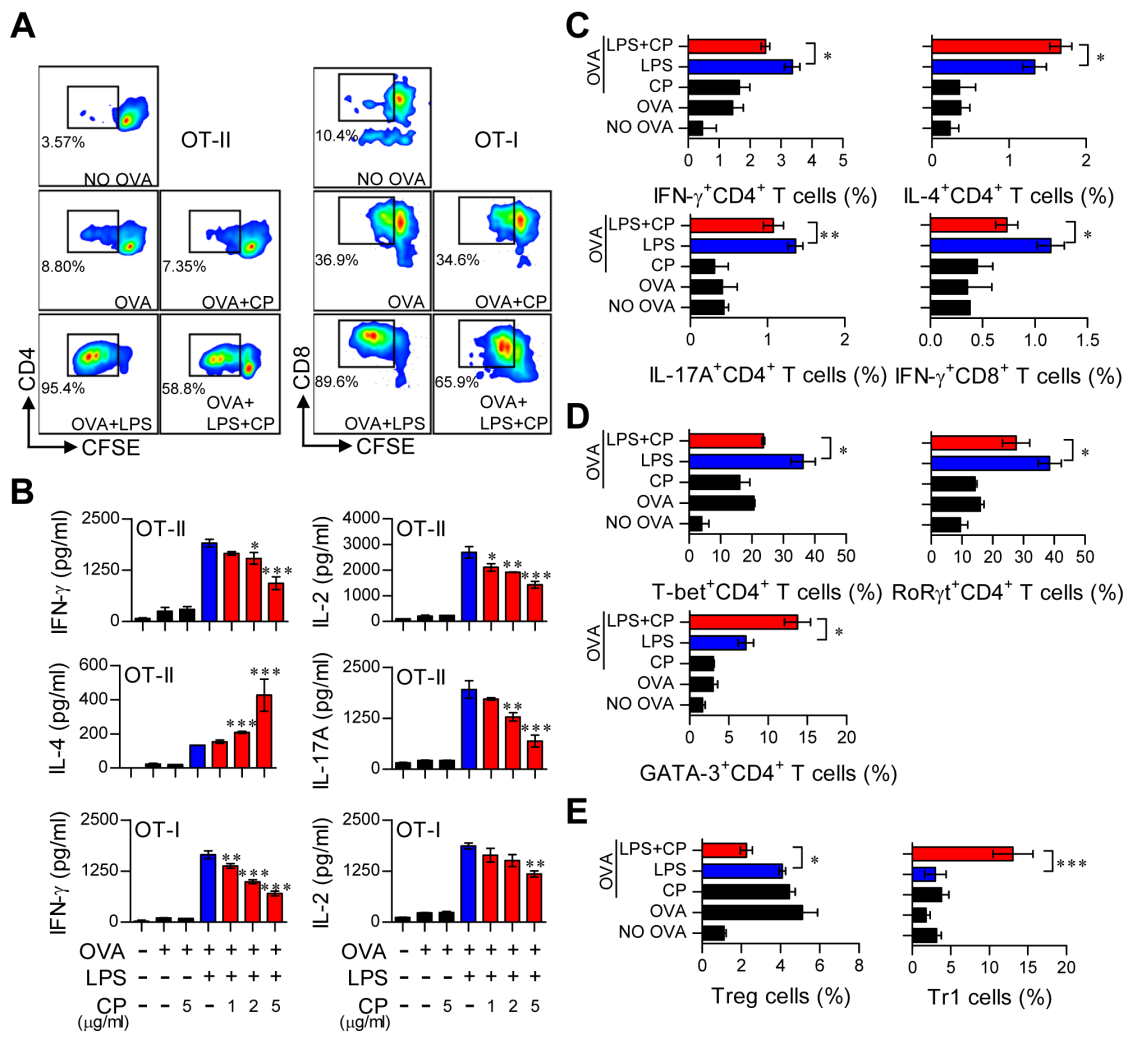
Cisplatin reduced the capacity of LPS-activated DCs to stimulate T cell proliferation and the Th1 response, polarized the Th2-type T cell response, and facilitated the generation of IL-10-producing Tr1 T cells Transgenic OVA-specific CD4^+^ T cells (OT-II) and transgenic OVA-specific CD8^+^ T cells (OT-I) were isolated; stained with CFSE; co-cultured for 96 h with DCs treated with LPS (100 ng/ml), cisplatin (5 μg/mL), or LPS and cisplatin; and then pulsed with OVA_323-339_ (1 μg/ml) for OVA-specific CD4^+^ T cells or OVA_257-264_ (1 μg/ml) for OVA-specific CD8^+^ T cells. **A.** The proliferation of CD4^+^ (left panel) and CD8^+^ T cells (right panel) was then assessed by flow cytometry. The T cell proliferation data are shown as one representative plot out of three independent experiments.**B.** Cytokine levels in the culture supernatants from CD4^+^ T cells (OT-II) and CD8^+^ T cells (OT-I) in the T cell proliferation experiments were measured by ELISA. **C.** The bar graphs show intracellular cytokine production (IFN-γ, IL-4, or IL-17A) in CD4^+^ andCD8^+^ T cells. **D.** The bar graphs show T cell transcription factor expression (T-bet for Th1, GATA-3 for Th2 and RoRγt for Th17) in CFSE-labeled CD4^+^ cells. **E.** The bar graphs show the percentages of Tregs and IL-10-producing Tr1 cells among CD4^+^ T cells. The values shown represent the mean ± SD (*n* = 4 samples) from one representative plot out of three independent experiments. *p < 0.05, **p < 0.01, and ***p < 0.001 compared with T cell/OVA_323-339_+LPS-pulsed DCs or T cells/OVA_257-264_ +LPS-pulsed DCs in the presence and absence of cisplatin.

Cisplatin treatment in LPS-activated DCs induced them to adopt a tolerogenic phenotype. Thus, we hypothesized that cisplatin treatment in LPS-activated DCs may reduce Th1 cell proliferation and alter T cell polarization. To precisely characterize the role of cisplatin in DC and T cell interactions, we performed a T cell proliferation assay for antigen-specific CD4^+^ and CD8^+^ T cells using OT-I T cell receptor (TCR) transgenic CD8^+^ T cells and OT-II TCR transgenic CD4^+^ T cells (Figure 6). LPS-treated or untreated DCs in the presence or absence of cisplatin were incubated with OVA_257-264_ or OVA_323-339_ peptides, and CFSE-labeled CD4^+^ and CD8^+^ T cells were co-cultured. As shown in Figure 6A, the cisplatin and LPS co-treated DCs reduced the proliferation of both naïve CD4^+^ and CD8^+^ T cells compared to the LPS-treated DCs. Furthermore, naïve CD4^+^ and CD8^+^ T cells primed with cisplatin and LPS co-treated DCs produced significantly lower levels of IFN-γ, IL-17A and IL-2 than those primed with LPS-activated DCs in a cisplatin dose-dependent manner (Figure 6B). These results were confirmed by intracellular staining (Figure 6C). Interestingly, IL-4, which is mainly produced by Th2-polarized T cells, was highly produced in CD4^+^ T cells primed with cisplatin and LPS co-treated DCs compared to those co-cultured with LPS-treated DCs in a cisplatin dose-dependent manner (Figure 6B and 6C).

We then investigated the differentiation and polarization of CD4^+^ T cells into Th1 cells, Th2 cells, Th17 cells, regulatory T cells (Treg) and T regulatory type 1 cells (Tr1) after priming with LPS-activated DCs in the presence or absence of cisplatin by analyzing transcription factor expression levels. Cisplatin and LPS co-treated DCs induced Th2 type cells (CD3^+^CD4^+^GATA-3^+^) and IL-10-producing Tr1 cells (CD3^+^CD4^+^CD25^−^CD49b^+^LAG-3^+^Foxp3^−^IL-10^+^) but decreased the numbers of Th1 (CD3^+^CD4^+^T-bet^+^), Th17 (CD3^+^CD4^+^RoRγt^+^) and Treg cells (CD3^+^CD4^+^CD25^+^CD127^−^Foxp3^+^) (Figure 6D and 6E). Additionally, cisplatin treatment produced the same proliferation and differentiation pattern for allogeneic T cells (Supplementary Figure S6). These results suggest that cisplatin induces the differentiation of Th2 and IL-10-producing Tr1 cells and decreases the proliferation of CD4^+^ and CD8^+^ T cells in response to LPS-activated DCs.

### DCs from IL-10^−/−^ mice do not induce the differentiation of Th2 and Tr1 cells

**Figure 7:**
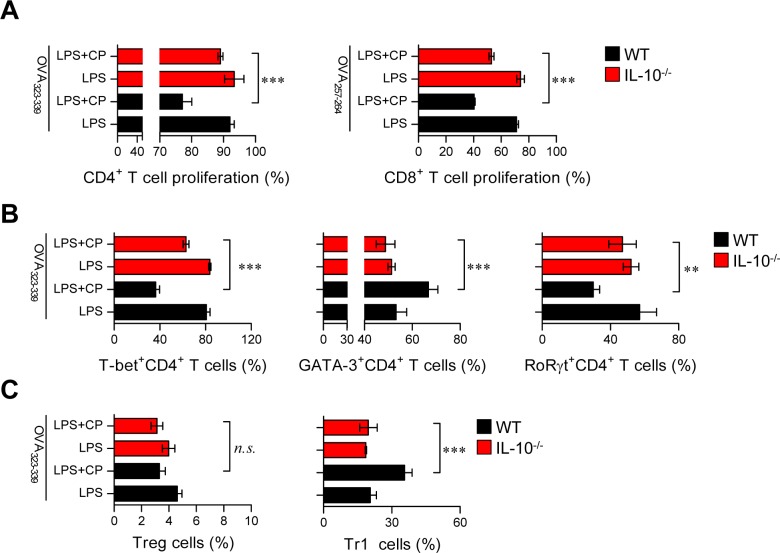
Absence of IL-10 in DCs restored the capacity of cisplatin/LPS-primed DCs to stimulate Th1-type T cell proliferation OVA-specific CD4^+^ and CD8^+^ T cells were isolated; stained with CFSE; co-cultured for 96 h with WT and IL-10^−/−^ DCs treated with LPS (100 ng/ml), cisplatin (5 μg/mL), or LPS and cisplatin; and then pulsed with OVA_323-339_ (1 μg/ml) for OVA-specific CD4^+^ T cells or OVA_257-264_ (1 μg/ml) for OVA-specific CD8^+^ T cells. **A.** The proliferation of CD4^+^ and CD8^+^ T cells was then assessed by flow cytometry. **B.** The bar graphs represent the T cell types, such as T-bet^+^CD4^+^, GATA-3^+^CD4^+^ and RoRγt^+^CD4^+^ cells, that were present among the CFSE-labeled CD4^+^ T cells. **C.** The bar graphs show the percentages of Treg cells and IL-10-producing Tr1 cells among the CD4^+^ T cells. All bar graphs show the mean ± SD (*n* = 4 samples) from one representative plot out of two independent experiments. **p < 0.01 and ***p < 0.001 when comparing WT DCs and IL-10^−/−^ DCs after co-treatment with LPS and cisplatin. Treatments with no significant effect are indicated as *n.s.*

IL-10-producing tolerogenic DCs potently induce the differentiation of naïve T cells towards Th2 and IL-10-producing Tr1 cells [22, 24]. In the same context as the results shown in Figure 6 and Supplementary Figure S6, we hypothesized that if IL-10 is a primary factor for inducing tolerogenic DCs and consequently polarizes CD4^+^ T cells towards Th2 and IL-10-producing Tr1 cells, the absence of IL-10 in DCs may prime the differentiation of CD4^+^ T cells towards Th1 and Th17 cells. Thus, we evaluated the capacity of IL-10^−/−^ DCs to induce T cell proliferation, differentiation, and polarization using the same experimental conditions described in Figure 6 (results shown in Figure 7). IL-10^−/—^derived DCs co-treated with cisplatin and LPS induced the proliferation of both CD4^+^ and CD8^+^ T cells (Figure 7A) and polarized naïve CD4^+^ T cells towards Th1 and Th17 cells instead of Th2 and Tr1 cells (Figure 7B) compared with DCs derived from wild-type (WT) mice. Finally, we confirmed that IL-10-producing Tr1-type T cells were not induced by IL-10^−/—^derived DCs in the presence of cisplatin (Figure 7C). Collectively, these results indicate that cisplatin functionally and phenotypically induces DCs towards an IL-10-producing tolerogenic phenotype upon TLR stimulation. These tolerogenic DCs consequently contribute to the development of Th2 and IL-10-producing Tr1 cells, suggesting that IL-10 is a primary factor responsible for these cascades.

### *In vivo* tolerogenic activity of cisplatin-treated DCs

**Figure 8:**
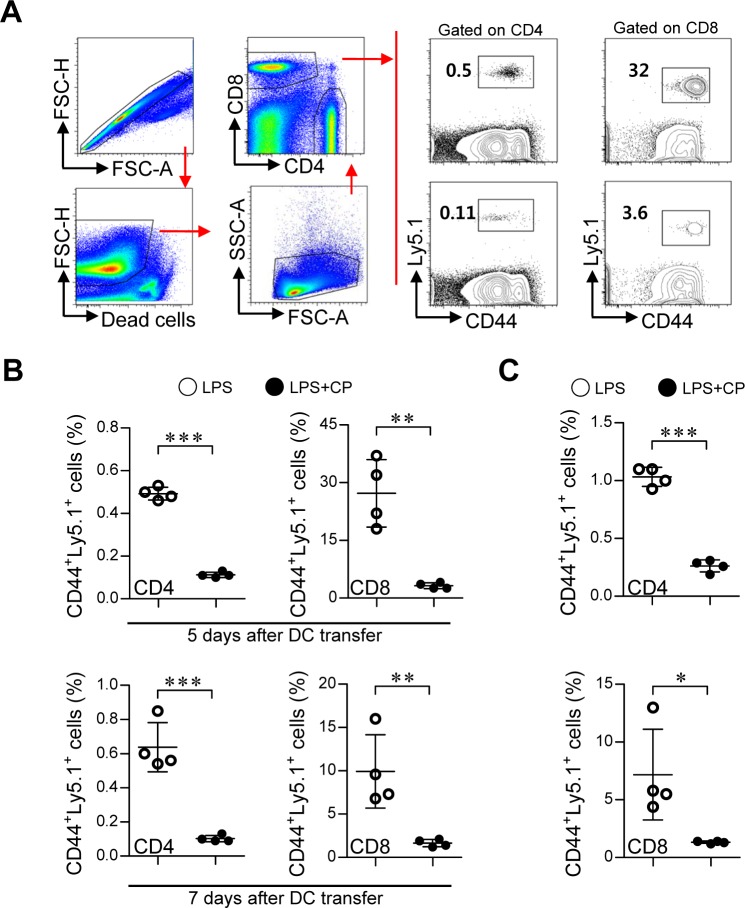
Cisplatin/LPS-primed DCs impaired T cell proliferation *in vivo* **A.**, **B.**, **C.** One day prior to injection with DCs (LPS/cisplatin-primed DC or LPS-primed DC), Ly5.2^+^ B6/J mice were injected with Ly5.1^+^ OT-I and OT-II T cells. At 5 and 7 days after DC injection, the frequencies of OVA-specific donor CD4^+^ and CD8^+^ T cells in PBMCs **A.**, **B.** and spleen cells **C.** were determined by flow cytometry. **A.** The gating strategy used to detect Ly5.1^+^CD44^+^CD4^+^ T cell and Ly5.1^+^CD44^+^CD8^+^ T cell populations present in the PBMCs at 5 days after injection with DCs. Doublets were excluded based on forward scatter area (FSC-A) *vs.* forward scatter height (FSC-H), and dead cells were excluded by gating out cells that stained positive with LIVE/DEAD viability dye. Lymphocytes were then gated based on their characteristic pattern of FSC, and SSC was excluded. Activated T cells (determined by the increase of CD44 expression) gated to show Ly5.1^+^CD44^+^CD4^+^ T cell and Ly5.1^+^CD44^+^CD8^+^ T cell populations within the collected PBMCs and spleen cells. **B.** Ly5.1^+^CD44^+^ T cell frequency was analyzed in the PBMCs at 5 and 7 days after injection with DCs. **C**. Ly5.1^+^CD44^+^ T cell frequency was analyzed in the spleen cells at 7 days after injection with DCs. The data are expressed as the means ± SD of 4 mice in each group. *p < 0.05, **p < 0.01, and ***p < 0.001 compared with the group injected with LPS-primed DCs. LPS: group injected with the LPS-primed DCs (○); LPS+CP: group injected with the LPS/cisplatin-primed DCs (•).

**Figure 9:**
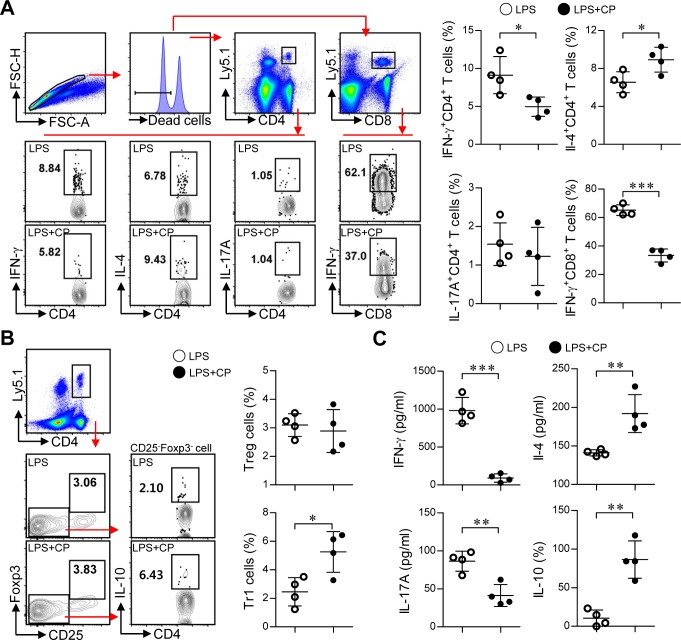
Cisplatin/LPS-primed DCs induced the generation of Th2-type cells and IL-10-producing Tr1 cells *in vivo* **A.**, **B.**, **C.** Spleen cells from each mouse group (i.e., mice injected with LPS-primed DCs and mice injected with Cisplatin/LPS-primed DCs) were stimulated with OVA_257-264_ and OVA_323-339_ for 12 h. The cells that had become stimulated with the OVA peptides were identified by intracellular cytokine staining of Ly5.1-expressing T cells. Further details regarding this *in vivo* experiment are provided in the *Materials and Methods* section. **A.** The frequencies of OVA-specific donor Th1 (Ly5.1^+^CD4^+^IFN-γ^+^), Th2 (Ly5.1^+^CD4^+^IL-4^+^), Th17 (Ly5.1^+^CD4^+^IL-17A^+^) and IFN-γ-producing CD8^+^ T cells (Ly5.1^+^CD8^+^IFN-γ^+^) were analyzed by flow cytometry. **B.** The frequencies of OVA-specific donor IL-10-producing Tr1 cells (Ly5.1^+^CD4^+^Foxp3^−^CD25^−^IL-10^+^) and Treg cells (Ly5.1^+^CD4^+^Foxp3^+^CD25^+^) were analyzed by flow cytometry. **C.** Culture supernatants obtained under the conditions described in the *Materials and Methods* section (*in vivo* experiment section) were harvested after 12 h, and IFN-γ, IL-4, IL-17A and IL-10 levels were analyzed by ELISA. The data are shown as the means ± SD of 4 mice per group. *p < 0.05 and***p < 0.001 compared with the group injected with LPS-primed DCs. LPS: group injected with LPS-primed DCs (○); LPS+CP: group injected with LPS/cisplatin-primed DCs (•).

Based on the above *in vitro* results, we next examined whether cisplatin and LPS co-treated DCs could suppress Th1-cell proliferation and induce the differentiation of Th2 and Tr1 cells *in vivo* (Figure 8 and Figure 9). Briefly, Ly5.1^+^ OT-I and OT-II T cells were transferred to Ly5.2^+^ recipient mice. The mice were intravenously injected with OVA protein-pulsed, LPS/cisplatin-primed DCs or OVA protein-pulsed, LPS-activated DCs one day after the T cell transfer. At 5 and 7 days after the DC injections, we evaluated the frequencies and types of Ly5.1^+^ T cells induced by the LPS/cisplatin-primed DCs (Figure 8 and Figure 9) using flow cytometry. As shown in Figure 8, significantly reduced frequencies of Ly5.1^+^CD44^+^CD4^+^ cells and Ly5.1^+^CD44^+^CD8^+^ cells were found in the peripheral blood mononuclear cell (PBMC) (Figure 8A and 8B) and splenocyte (Figure 8C) populations isolated from the LPS/cisplatin-primed DC group compared to the LPS-activated DC group. Importantly, the spleen cells isolated from the LPS/cisplatin-primed DCs group showed significantly reduced frequencies of Th1-type cells (IFN-γ-producing Ly5.1^+^CD4^+^ T cells) and IFN-γ-producing Ly5.1^+^CD8^+^ T cells compared with the LPS-primed DCs group in response to stimulation with OVA peptides (Figure 9A). Additionally, the LPS/cisplatin-primed DCs group showed significant induction of Th2-type cells (IL-4-producing Ly5.1^+^CD4^+^ T cells) and Tr1 cells (IL-10-producing Ly5.1^+^CD25^−^Foxp3^−^CD4^+^ T cells) (Figure 9A and 9B). Culture supernatants containing secreted cytokines were collected from the splenocytes following stimulation with OVA peptides and analyzed by ELISA. The results showed that the LPS/cisplatin-primed DCs group robustly secreted both Th2-related cytokines and Tr1-related cytokines such as IL-4 and IL-10, whereas they secreted significantly low levels of Th1-related (IFN-γ) and Th17-related (IL-17A) cytokines (Figure 9C). These results indicate that cisplatin-treated DCs have *in vivo* tolerogenic activity by inducing the differentiation of Th2 and IL-10-producing Tr1 cells and decreasing the proliferation of CD4^+^ and CD8^+^ T cells.

## DISCUSSION

Although cisplatin is a common treatment for a broad spectrum of cancers, few studies have evaluated the effect of cisplatin treatment on the immune system. In the present study, we described the molecular mechanisms that are required for the immunosuppressive effect of cisplatin, including the phenotypic alteration of DCs and their interactions with T cells, thereby improving our understanding of the immunological aspects of this cancer chemotherapy. Cisplatin induced IL-10-producing tolerogenic DCs upon TLR agonist treatment by activating the p38 MAPK signaling pathway, and these cisplatin-induced tolerogenic DCs promoted the generation of two distinct T cell subsets *in vivo* as well as *in vitro*: Th2-type T-cells and IL-10-producing Tr1 cells.

Several cancer drugs are also used as immunosuppressants for the treatment of severe systemic autoimmune diseases. Interestingly, cisplatin has shown therapeutic potential for treating inflammation-mediated diseases under different pathophysiological conditions [25, 26]. For example, cisplatin was shown to ameliorate the symptoms of excessive inflammation-mediated diseases, such as experimental autoimmune encephalomyelitis (EAE), *in vivo* [25]. EAE is a prototypic Th1-mediated autoimmune inflammatory disease, and Th17 cells are assumed to be crucial effector cells that drive central nervous system (CNS) inflammation and damage [25, 27]. In addition, low-dose cisplatin administration in a murine cecal ligation and puncture-induced polymicrobial sepsis model was found to attenuate inflammation and lethality [26]. Thus, cisplatin might be considered a candidate drug for the control of inflammatory and autoimmune processes. However, it remains unclear how cisplatin ameliorates excessive inflammation. Our findings help explain the immunomodulatory activities of cisplatin observed in these disease models.

The nature of DCs determines the quality of T cell responses and the establishment of either immunity or tolerance. In a previous study, Dijkgraaf et al. showed that cisplatin treatment increased the PGE2-secreting potency of tumor cells to promote monocyte differentiation into IL-10-producing M2 macrophages. These authors proposed a chemotherapy-induced increase in tumor-promoting M2 macrophages may provide an indirect mechanism for chemoresistance and that concomitant therapy with COX inhibitors might enhance the anti-tumor effect of cisplatin [28]. Similarly, our data showed that cisplatin directly skewed the phenotype and function of DCs towards IL-10-producing-tolerogenic DCs upon TLR ligand stimulation (Figure 2 and Supplementary Figure S3). However, the COX-2-PGE2 pathway did not induce tolerogenic DCs and IL-10 production in our study (Supplementary Figure S4). In addition, MyD88 or TRIF deficiency abrogated IL-10 production in cisplatin and LPS co-treated DCs, indicating that TLR signaling is a prerequisite for the generation of IL-10-producing tolerogenic DCs (Supplementary Figure S5). Although it remains debatable whether the clinical success of cisplatin relies primarily on its ability to trigger apoptosis, cisplatin induces cell death mainly *via* apoptosis and eventually secondary necrosis, and endogenous TLR ligands are released during cell death. Along with the recognition of microbial TLR ligands called pathogen-associated molecular patterns (PAMPs), an increasing number of endogenous TLR stimulators have been reported. These endogenous molecules activate TLR signaling and are involved in many pathological processes. For example, HMGB1 and heat shock proteins are either passively released from injured/inflamed tissues and dying cells or actively secreted by activated cells under pathological conditions. Furthermore, even apoptotic cells may release these endogenous TLR ligands [29]. In addition, a single endogenous molecule, such as HMGB1, has the potential to interact with several TLRs. Thus, these endogenous TLR ligands may participate in tumor progression. In addition, evidence indicates that dying tumor cells release endogenous TLR ligands [30–32]. In fact, HMGB1 released following chemotherapy-induced cell death activates DCs *via* the TLR4-MyD88 axis, which leads to the induction of anti-tumor T-cell immunity [30].

IL-10-producing tolerogenic DCs are potent inducers of naïve T cell differentiation towards Th2 and IL-10-producing Tr1 cells [33]. During sterile inflammation caused by the exposure of normal cells to anti-cancer drugs, an enormous number of cells undergo apoptosis and/or necrosis in different organs and tissues. These dying cells release endogenous ligands, such as intracellular proteins and nucleic acids, which can bind to pattern recognition receptors, particularly those on tissue-resident innate immune cells such as DCs [34]. In response to activation signals, these cells can inhibit tissue damage by producing anti-inflammatory factors such as IL-10 [35]. However, because the large amounts of IL-10 that are present in the tumor microenvironment play a major role in suppressing the anti-tumor immune response, strategies to break tolerance by neutralizing the function of IL-10 are important for tumor treatment. In a recent study, renal DCs attenuated cisplatin-induced kidney inflammation by producing a high level of IL-10 in response to cisplatin treatment [36]. In addition, *in vivo* cisplatin treatment directly caused a 10-fold increase of IL-10 production in renal DCs, but not in whole kidneys, indicating that DCs are the major source of IL-10 after cisplatin treatment [37]. However, little is known about the signaling pathways through which cisplatin triggers the generation of tolerogenic DCs after encountering inflammatory environments and their roles in preserving the tolerogenic properties of DCs. Our current study is the first to perform a detailed analysis of the molecular mechanisms responsible for the induction of tolerogenic DCs by cisplatin in the inflammatory environment. We found that a large amount of IL-10 was produced after cisplatin treatment and that this production was stimulated through the MAPK and NF-κB signaling pathways (Figure 3). Without an IL-10 signal, cisplatin-treated DCs did not differentiate into tolerogenic DCs, indicating that induction of tolerogenic DCs by cisplatin requires IL-10 production (Figure 4 and Figure 5). There are several studies showing that p38 MAPK activation in immunosuppressive CD25^−^ regulatory T cells [38] and tolerogenic DCs [39, 40] is a central pathway to induce IL-10 production. Our finding that cisplatin activates p38 MAPK may explain why cisplatin treatment leads to the generation of IL-10-producing tolerogenic DCs. Moreover, the inhibition of p38 MAPK signaling in DCs has been suggested as a way to break immunological tolerance and redirect T cell responses to enhance anti-tumor immunity. The importance of IL-10 production by DCs in the induction of tolerance has been demonstrated, as DCs genetically modified to express IL-10 exhibit suppressive effects in models of alloreactivity and autoimmunity [41, 42]. Jarnicki et al. further demonstrated that the inhibition of p38 MAPK-dependent IL-10 secretion by DCs led to stronger Th1 responses and reduced Tr1 induction [39], indicating that the immune tolerance that is induced by cisplatin can be overcome by inhibiting p38 signaling in DCs. Thus, p38 is an important therapeutic target and provides a mechanism to enhance the efficacy of TLR agonists as vaccine adjuvants together with anti-cancer drugs.

One provocative finding of our study was the induction of IL-10-producing CD3^+^CD4^+^LAG-3^+^CD49b^+^CD25^−^Foxp3^−^ Tr1 cells by cisplatin/LPS-primed DCs both *in vitro* and *in vivo* (Figure 6, Figure 8, Figure 9 and Supplementary Figure S6). Tr1 cells are induced in the periphery and play a pivotal role in promoting and maintaining tolerance. IL-10-secreting Tr1 cells were first defined by their specific cytokine production profile, which includes the secretion of high levels of IL-10 and transforming growth factor-b (TGF-b), and by their ability to suppress antigen-specific effector T cell responses *via* a cytokine-dependent mechanism [43]. In other words, the induction of IL-10-secreting T-cell populations with regulatory activity is IL-10 dependent. In contrast to the naturally occurring Tregs that emerge directly from the thymus, Tr1 cells are induced by antigen stimulation *via* an IL-10-dependent process *in vitro* and *in vivo*. Thus, specialized IL-10-producing DCs may play a key role in this process. However, we cannot completely exclude the secondary effect of IL-10 produced from T cells in accelerating the differentiation of Tr1 cells, indicating that further study of the induction of Tr1 differentiation *via* interactions between IL-10-producing DCs and IL-10-producing T cells is required.

Many studies have suggested that chemotherapy resistance is associated with a strong immune-suppressive tumor microenvironment [44], and our data suggest a possible underlying mechanism for systemic tumor progression in cases of relapse after chemotherapy. Although current treatment protocols, including surgery and chemotherapy, can achieve local control of a tumor, patients who relapse after long-term cisplatin treatment may develop systemic disease due to the cisplatin-induced generation of tolerogenic DCs, which limit the Th1 immune response.

In summary, we demonstrated that cisplatin reprograms TLR agonist-induced activation of DCs *via* p38 MAPK-induced IL-10 production to generate tolerogenic DCs, which in turn promote the differentiation of naïve CD4^+^ T cells into IL-10-producing Tr1 cells. A better understanding of the different effects of cisplatin on immune cells compared to cancer cells may lead to the design of more successful anti-tumor drugs and may also provide new therapeutic strategies based on cisplatin-induced immunological modulation.

## MATERIALS AND METHODS

### Experimental animals

Specific pathogen-free, female, 6- to 8-week-old C57BL/6 (H-2Kb and I-Ab), BALB/c (H-2Kd and I-Ad), C57BL/6 IL-10^−/−^, C57BL/6 MyD88^−/−^, C57BL/6 TRIF^−/−^, C57BL/6 OT-I and OT-II OVA specific T cell receptor (TCR) transgenic mice were purchased from The Jackson Laboratory (Bar Harbor, ME, USA).

### Antibodies and reagents

Recombinant mouse granulocyte-macrophage colony stimulating factor (GM-CSF) was purchased from R&D Systems (Minneapolis, MN, USA). 3-(4, 5-dimethylthiazol-2-yl)-2, 5-diphenyl-tetrazolium bromide (MTT) was purchased from Sigma-Aldrich Chemical Co. (St Louis, MO, USA). Pam3CSK4, Poly I:C, lipopolysaccharide (LPS, from *Escherichia coli* O111:B4), Imiquimod (R837) and ODN1826 were purchased from Invivogen (San Diego, CA, USA). The OT-I peptide (OVA_257-264_, SIINFEKL) and OT-II peptide (OVA_323-339_, ISQAVHAAHAEINEAGR) were synthesized by Abfrontier (Seoul, Korea). Anti-phosphorylated ERK1/2, JNK, and p38 mouse monoclonal antibodies (mAbs) and anti-ERK1/2, JNK, p38 mAbs were obtained from Santa Cruz Biotechnology, Inc. (Santa Cruz, CA, USA). A horseradish peroxidase (HRP)-conjugated anti-mouse Ab and a HRP-conjugated anti-rabbit Ab were obtained from Calbiochem (San Diego, CA, USA). PE-conjugated mAbs to CD80, MHC-I, IFN-γ, IL-4, GATA-3 and Foxp3; PE-Cy7-conjugated mAbs to CD11c, CD25 and T-bet; PerCP-Cy5.5-conjugated mAbs to CD223 (LAG-3), CD4 and CD8; APC-conjugated mAbs to CD86, MHC-II, CD49b, IL-10, IL-12p70, TNF-α and RoRγt; APC-Cy7-conjugated CD127 mAb; BV510-conjugated IL-10 mAb; APC-Cy7-conjugated IL-17A mAb; and a FITC-conjugated mAb to CD4 were purchased from eBioscience (San Diego, CA, USA). TNF-α, IL-2, IL-4, Th17A and IFN-γ ELISA kits were obtained from eBioscience. Anti-Armenian hamster IgG, anti-rat IgG2a K, anti-rat IgG2b, anti-mouse IgG1 K and anti-mouse IgG2a isotype controls were purchased from eBioscience. Neutralizing anti-IL-10 mAb (JESS-2A5) and isotype control rat IgG1 (HRPN) were purchased from Bio X Cell (West Lebanon, NH, USA).

### Differentiation of BMDCs

Whole bone marrow cells isolated from C57BL/6 mice were incubated at 37°C in a 5% CO_2_ atmosphere using RPMI 1640 medium supplemented with 100 U/mL penicillin/streptomycin (Lonza, Basel, Switzerland), 10% fetal bovine serum (Lonza), 50 μM mercaptoethanol (Lonza), 0.1 mM nonessential amino acids (Lonza), 1 mM sodium pyruvate (Sigma Chemical Co., St. Louis, MO, USA) and GM-CSF (20 ng/mL). On day 8, over 80% of the non-adherent cells expressed CD11c. To obtain highly purified populations ( > 95% cell purity), in some experiments, the DCs were labeled with a bead-conjugated anti-CD11c mAb (Miltenyi Biotec, Bergisch Gladbach, Germany) followed by positive selection on paramagnetic columns (LS columns; Miltenyi Biotec) according to the manufacturer's instructions.

### Annexin V and propidium iodide (PI) assay using flow cytometry

After 8 days of culture, BMDCs were treated with LPS and/or cisplatin. After 18 h of treatment, harvested DCs were washed with PBS and stained using the Annexin V-PI Apoptosis Detection kit (BD Biosciences) protocol.

### Cell survival assay

Cell viability was measured by MTT assay. MTT was dissolved in PBS at a final concentration of 0.5 mg/ml. A 100-μl aliquot of the MTT solution was added to each well of a 96-well plate, and the plate was incubated at 37°C for 4 h. The resultant formazan crystals were dissolved in DMSO, and the OD570 nm values of each well were measured using a microplate reader.

### Analysis of DC surface molecule expression using flow cytometry

On day 8, BMDCs were harvested, washed once, resuspended at 1×10^6^ cells/ml, and treated with the following stimuli for 18 h: medium alone (CON; control), LPS, cisplatin, or LPS and cisplatin. The cells were first blocked with 10% (v/v) normal goat serum for 15 min at 4°C and then stained with anti-MHC-I, anti-MHC-II, anti-CD80, anti-CD86, and anti-CD11c mAbs for 30 min at 4°C. Finally, the stained cells were analyzed using an LSRII flow cytometer (Becton Dickinson, San Jose, CA, USA) and FlowJo software (Tree Star, Inc., Ashland, OR).

### ELISA

To assess cytokine production, supernatants were collected 18 h after treatment of DCs and stored at −70°C. The amounts of TNF-α, IL-12p70, IL-10, IFN-γ, IL-2, IL-4 and IL-17A were measured by sandwich ELISA kits using commercially available pairs of antibodies and standards according to the manufacturer's protocol.

### Intracellular cytokine staining

Cells were first blocked with 10% (v/v) normal goat serum for 15 min at 4°C and then stained with CD11c, CD4 and CD8 mAbs for 30 min at 4°C. Cells stained with the appropriate isotype-matched IgG were used as negative controls. The cells were fixed and permeabilized with a Cytofix/Cytoperm kit (BD Biosciences or eBioscience) according to the manufacturer's instructions. Next, anti-IL-12p70, anti-IL-10, anti-TNF-α, anti-IFN-γ, anti-IL-4, anti-IL-17A, anti-T-bet, anti-GATA-3 and anti-RoRγt mAbs were stained with fluorescein-conjugated secondary Abs in a permeation buffer.

### Antigen uptake assay

BMDCs were equilibrated at 37°C or 4°C for 45 min and then pulsed with fluorescein-conjugated dextran (40,000 Da) from Sigma-Aldrich (St. Louis, MO) at a concentration of 1 mg/ml. After several washes with cold phosphate-buffered solution, the cells were stained with a PE-Cy7-conjugated anti-CD11c mAb and then measured with a FACS LSRII to reveal antigen uptake. Nonspecific binding of dextran to DCs was determined by incubating DCs with FITC-conjugated dextran at 4°C, and the resulting background value was subtracted from the specific binding values.

### Immunoblotting analyses

After stimulation with LPS, cisplatin, or LPS and cisplatin, DCs were lysed in 100 μl lysis buffer containing 50 mM Tris-HCl (pH 7.5), 150 mM NaCl, 1% Triton-X100, 1 mM EDTA, 50 mM NaF, 30 mM Na4PO7, 1 mM phenylmethanesulfonyl fluoride, 2 μg/ml aprotinin, and 1 mM pervanadate. Whole-cell lysate samples were resolved on SDS-polyacrylamide gels and then transferred onto nitrocellulose membranes for 1 h at 60 V. The membranes were blocked in 5% skim milk and incubated with primary Abs for 2 h, followed by incubation with HRP-conjugated secondary Abs for 1 h at room temperature. Target protein epitopes were labeled with primary Abs against total MAPKs and IκB-α (1:1,000 diluted in blocking solution) and polyclonal Abs against p-ERK 1/2, p-JNK 1/2, p-p38, p-IκB-α and p65 (1:5,000, 1:2,000, 1:5,000, 1:2,000 and 1:3,000 dilutions, respectively), and the results were visualized using an ECL Advance Western Blotting Detection kit (GE Healthcare, Little Chalfont, U.K.).

### Nuclear extract preparation

BMDCs were treated with 150 μl lysis buffer [10 mM HEPES (pH 7.9), 10 mM KCl, 0.1 mM EDTA, 0.5% Nonidet P-40, 1 mM KCL, 1 mM dithiothreitol, 0.5 mM PMSF] on ice for 15 min. After centrifugation at 3,000 rpm for 3 min, the pellet was resuspended in 100 μl extraction buffer [10 mM HEPES (pH 7.9), 400 mM NaCl, 1 mM EDTA, 1 mM DTT, 1 mM PMSF] and incubated on ice for 30 min, followed by centrifugation at 12,000 rpm for 5 min. The supernatant containing nuclear extracts was collected.

### Treatment of DCs with pharmacological inhibitors

All pharmacological inhibitors were obtained from Calbiochem; reconstituted in sterile, cell-culture grade DMSO; and used at the following concentrations: SP600125 (1, 5 and 10 μM), SB203580 (1, 5 and 10 μM) and Bay-117082 (5, 10 and 20 μM). A 0.1% DMSO solution was used as the vehicle control. For the experiments with inhibitors, cells were treated with pharmacological inhibitors for 1 h prior to treatment with a combination of LPS and cisplatin for 18 h.

### Analysis of T cell differentiation and proliferation both *in vitro* and *in vivo*

T cell proliferation and differentiation were assayed *in vitro* and *in vivo* using a modified previously described protocol [45, 46]. Responder T cells, which participate in naive T cell reactions, were isolated using a MACS column from total mononuclear cells prepared from OT-I, OT-II and naive BALB/c mice. For the *in vitro* experiment, OVA-specific CD8^+^ and CD4^+^ T cells, both responders, were obtained from the splenocytes of OT-I and OT-II mice, respectively. These T cells were stained with 1 μM carboxyfluorescein diacetate succinimidyl ester (CFSE; Invitrogen). DCs (2×10^5^ cells/well) treated with OVA peptide in the presence of LPS (100 ng/ml), cisplatin or a combination of LPS and cisplatin for 18 h were co-cultured with CFSE-stained CD8^+^ and CD4^+^ T cells (2×10^6^) at a DC:T-cell ratio of 1:10. Naive BALB/c (allogeneic mixed lymphocyte reaction (MLR)) T cells (2×10^6^) were co-cultured with DCs (2×10^5^ cells/well) treated with LPS, cisplatin or LPS and cisplatin. On day 3 or day 4 of co-culture, T cells were stained using an intracellular staining method. Culture supernatants were harvested, and IFN-γ, IL-2, IL-4 and IL-17A levels were measured by ELISA.

For the *in vivo* experiment, Ly5.1^+^ OT-I and OT-II T cells (5 × 10^5^ cells per population) were transferred intravenously into the lateral tail veins of Ly5.2^+^ recipient mice. The next day, BMDCs from Ly5.2^+^ recipient mice were pulsed with ovalbumin protein (10 μg/ml) for 2 h, and then the cells were washed with wash buffer (RPMI 1640 plus 2% FBS). After washing twice with wash buffer, the DCs were stimulated by LPS with or without cisplatin for 18 h. These DCs (5 × 10^6^ cells) were transferred intravenously into the lateral tail veins of Ly5.2^+^ recipient mice. At day 5 and day 7 after the DC transfer, PBMCs were isolated from blood samples collected from each mouse, as previously described [47]. To analyze the populations of Ly5.1^+^ OT-I and OT-II T cells, single-cell suspensions of PBMCs and splenocytes from each mouse were stained with anti-CD4, anti-CD8α, anti-CD44, and anti-Ly5.1 mAbs. Dead cells were excluded using a Live/Dead Fixable Dead Cell Stain kit (Invitrogen). Data were collected on a FACS Canto II (BD) and a FACSverse (BD) and analyzed using FlowJo software (TreeStar). Additionally, single-cell suspensions prepared from the spleens of each group were stimulated with OVA_257-264_ (0.2 μg/ml) and OVA_323-339_ (5 μg/ml) for 12 h at 37°C in the presence of GolgiPlug 7 days after DC transfer. At 12 h after stimulation, the types and populations of T cells (Ly5.1^+^ T cells) present were analyzed by intercellular staining and flow cytometry. Supernatants were harvested, and IFN-γ, IL-4, IL-17A and IL-10 levels were measured by ELISA.

### Statistical analysis

Significant differences between samples were determined with Tukey's multiple comparison test and unpaired *t*-tests using statistical software (GraphPad Prism Software, version 5; GraphPad Software, San Diego, CA). The data in the graphs are expressed as the means. *p < 0.05, **p < 0.01 and ***p < 0.001 were considered statistically significant.

## SUPPLEMENTARY MATERIAL FIGURES


