# Supplementary material for: Cisplatin induces tolerogenic dendritic cells in response to TLR agonists via the abundant production of IL-10, thereby promoting Th2- and Tr1-biased T-cell immunity
Source: Oncotarget. 2016 May 9;7(23):33765–82. doi: 10.18632/oncotarget.9260 (PMC5085117; doi:10.18632/oncotarget.9260)
Supplement: Supplementary file 1 [file oncotarget-07-33765-s001.pdf]

Cisplatin induces tolerogenic dendritic cells in response to TLR agonists via the abundant production of IL-10, thereby promoting Th2- and Tr1-biased T-cell immunity

Supplementary Material

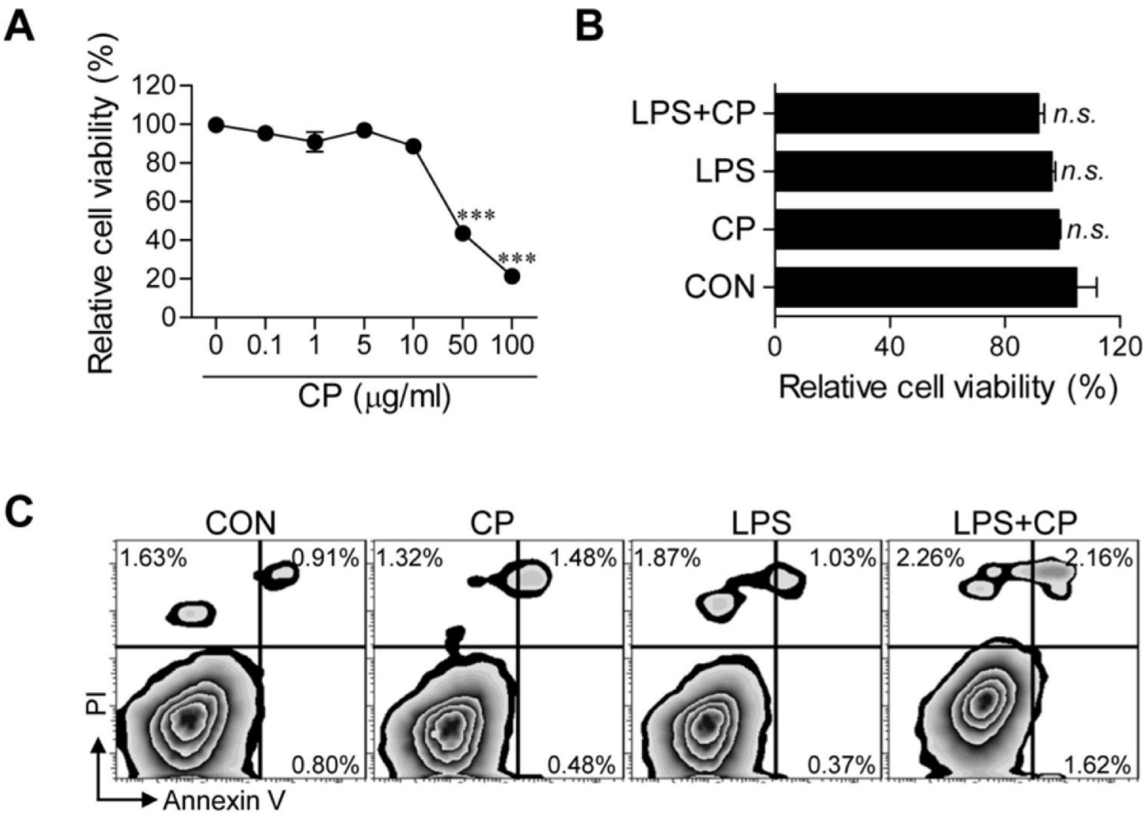

Supplementary Figure S1: Effect of cisplatin on cell viability. **A, B.** Cell viability was assessed using an MTT assay. **A.** BMDCs were treated with increasing concentrations (0, 0.1, 1, 5, 10, 50 and 100 μg/mL) of cisplatin on day 8 of culture and harvested 18 h later to determine the concentration that does not cause cell death. **B**, **C.** BMDCs were treated with LPS (100 ng/ml), cisplatin (10 μg/mL), or LPS with cisplatin, and cell toxicity was determined using an MTT assay and Annexin V/PI staining. The results are representative of three independent experiments. ***$p < 0.001$; *n.s.*, no significant effect.

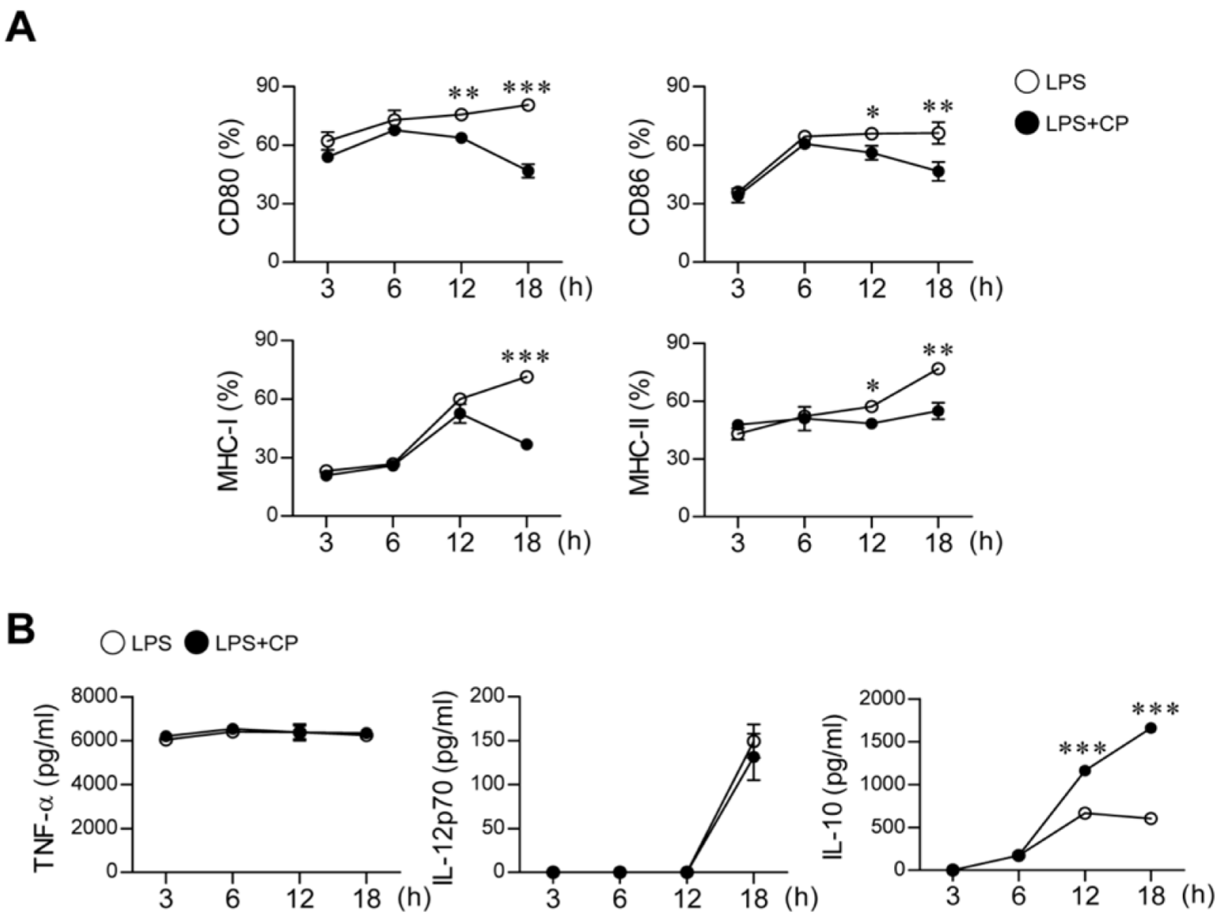

Supplementary Figure S2: Cisplatin induced IL-10 production and reduced the expression of surface molecules in a time-dependent manner. A, B. BMDCs were activated with LPS (100 ng/ml) with or without cisplatin (5 μg/mL), and surface molecule expression patterns and cytokine release kinetics were analyzed after various time points (3, 6, 12 and 24 h). **A.** DCs were stained with anti-CD80, anti-CD86, anti-MHC class I, or anti-MHC class II mAbs and analyzed for the expression of surface markers. **B.** The amounts of TNF-α, IL-12p70, and IL-10 in the culture medium were measured by ELISA. The results are representative of three independent experiments. **$p < 0.01$ and ***$p < 0.001$ compared to DCs treated with LPS alone.

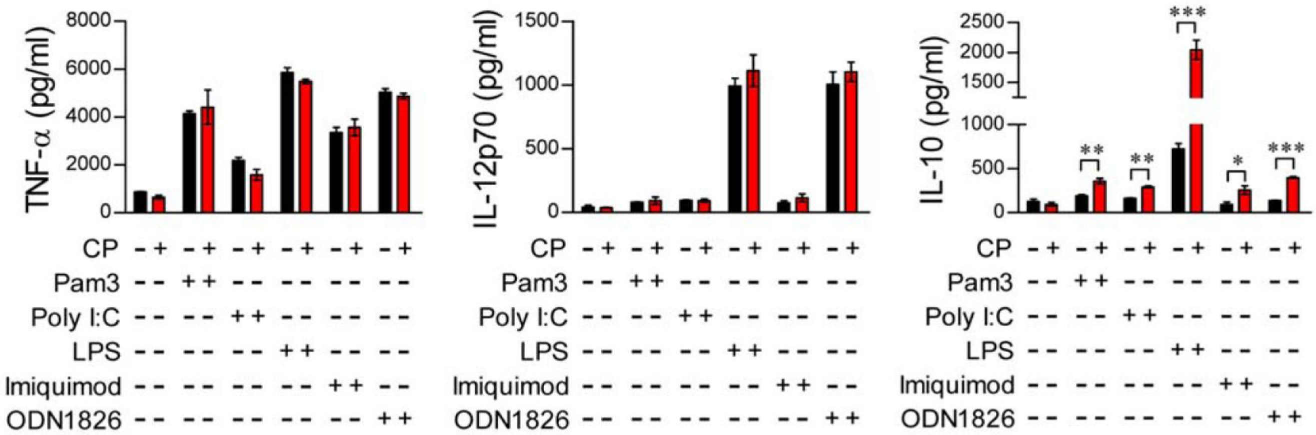

Supplementary Figure S3: IL-10 production is increased by cisplatin in association with TLR agonists. BMDCs were treated with TLR agonists (TLR2, TLR3, TLR4, TLR7 and TLR9) in the absence or presence of cisplatin (5 μg/ml) for 18 h. The amount of IL-10 in the culture media was measured by ELISA. All data are expressed as the mean ± SD (*n* = 3 samples). The results are representative of two independent experiments. **p* < 0.05, ***p* < 0.01, and ****p* < 0.001.

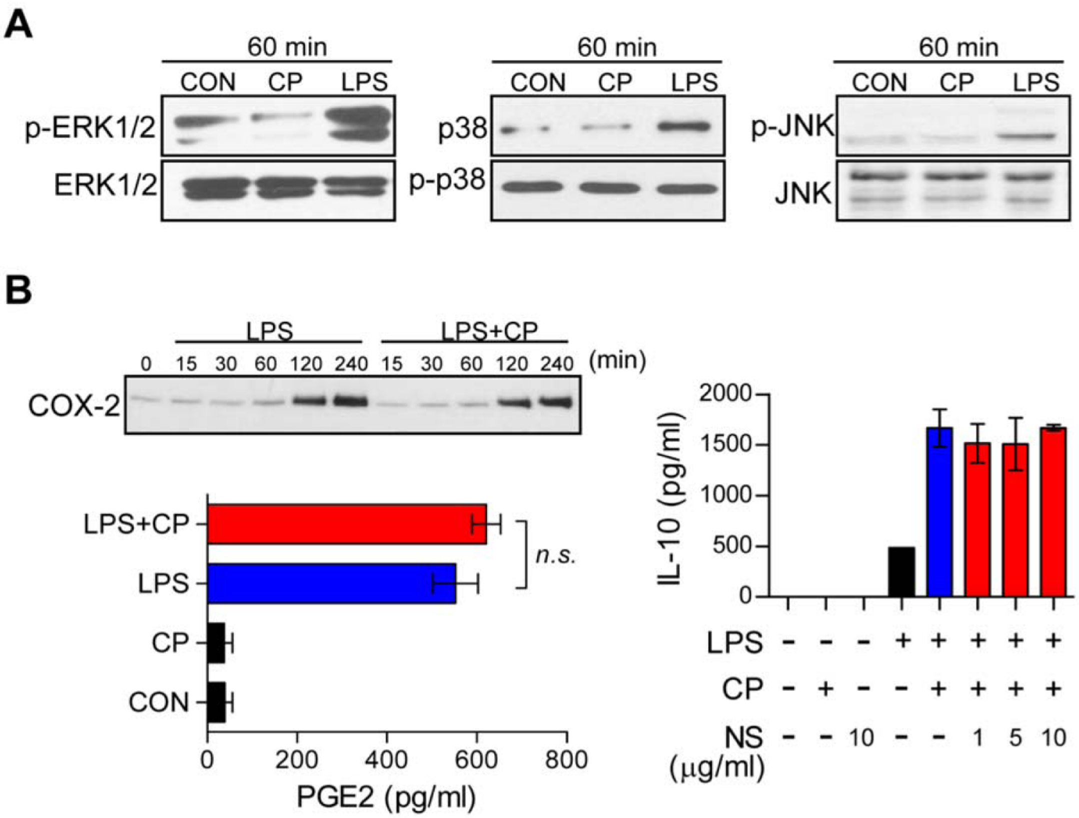

Supplementary Figure S4: Effect of cisplatin on MAPK activity, COX-2 expression and PGE2 secretion. A. For MAPK activation analyses, DCs were pretreated with cisplatin (5 µg/ml) for 60 min. The data shown are from one representative plot out of two independent experiments. **B.** BMDCs were treated with LPS (100 ng/ml) in the absence or presence of cisplatin for various times. COX-2 expression was measured by Western blotting of total cell lysates (left top panel). Secreted PGE2 levels in the supernatants of DCs treated with LPS, cisplatin or LPS and cisplatin for 18 h (left bottom panel) were measured by ELISA. DCs were treated with pharmacological inhibitors of COX-2 (non-steroidal anti-inflammatory drug, NS) or DMSO (vehicle control) for 1 h prior to treatment with LPS in the absence or presence of cisplatin for 18 h (right panel). The amount of IL-10 in the culture medium was measured by ELISA. All data are expressed as the mean ± SD (n = 3 samples). The results are representative of two independent experiments. *n.s.*, no significant effect.

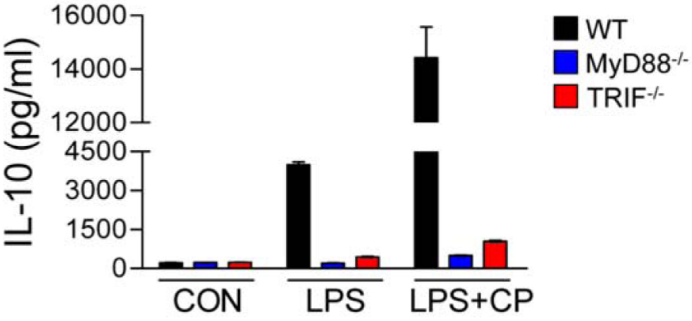

Supplementary Figure S5: Effects of MyD88 and TRIF signaling on IL-10 secretion. BMDCs derived from WT, MyD88$^{-/-}$ and TRIF$^{-/-}$ mice were treated with LPS (100 ng/ml), cisplatin (5 μg/mL), or LPS with cisplatin for 18 h. The amount of IL-10 in the culture supernatant was measured by ELISA. The data are expressed as the mean ± SD (n = 3 samples) and are representative of two independent experiments. ***$p < 0.001$.

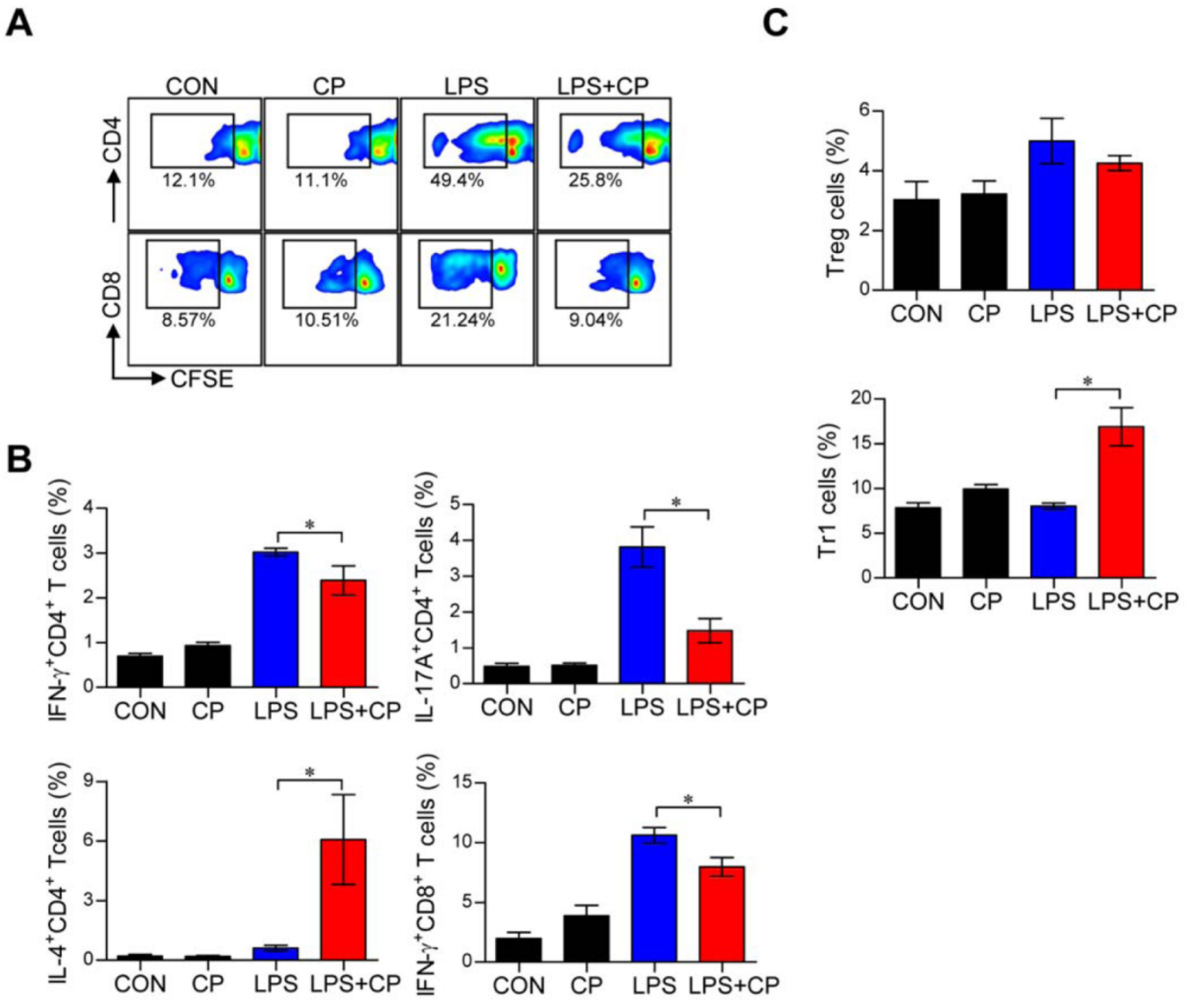

Supplementary Figure S6: Effects of cisplatin on the proliferation and differentiation of allogenic T cells induced by LPS-activated DCs. T cells were isolated from BALB/c mice, stained with CFSE, and co-cultured for 96 h with DCs treated with LPS (100 ng/ml), cisplatin (5 μg/mL), or LPS and cisplatin, and T cell proliferation was analyzed by flow cytometry. **A.** T cell proliferation measured from one representative plot out of three independent experiments. **B.** Analysis of intracellular cytokine production (IFN-γ, IL-4, and IL-17A) in CD4$^+$ T cells co-cultured with activated DCs. **C.** CD4$^+$ T cells were measured for Treg and IL-10-producing Tr1 cell induction in the presence and absence of cisplatin. All bar graphs are expressed as the mean ± SD (n = 3 samples). The results are representative of two independent experiments. *$p < 0.05$ compared with T cells/LPS-pulsed DCs.

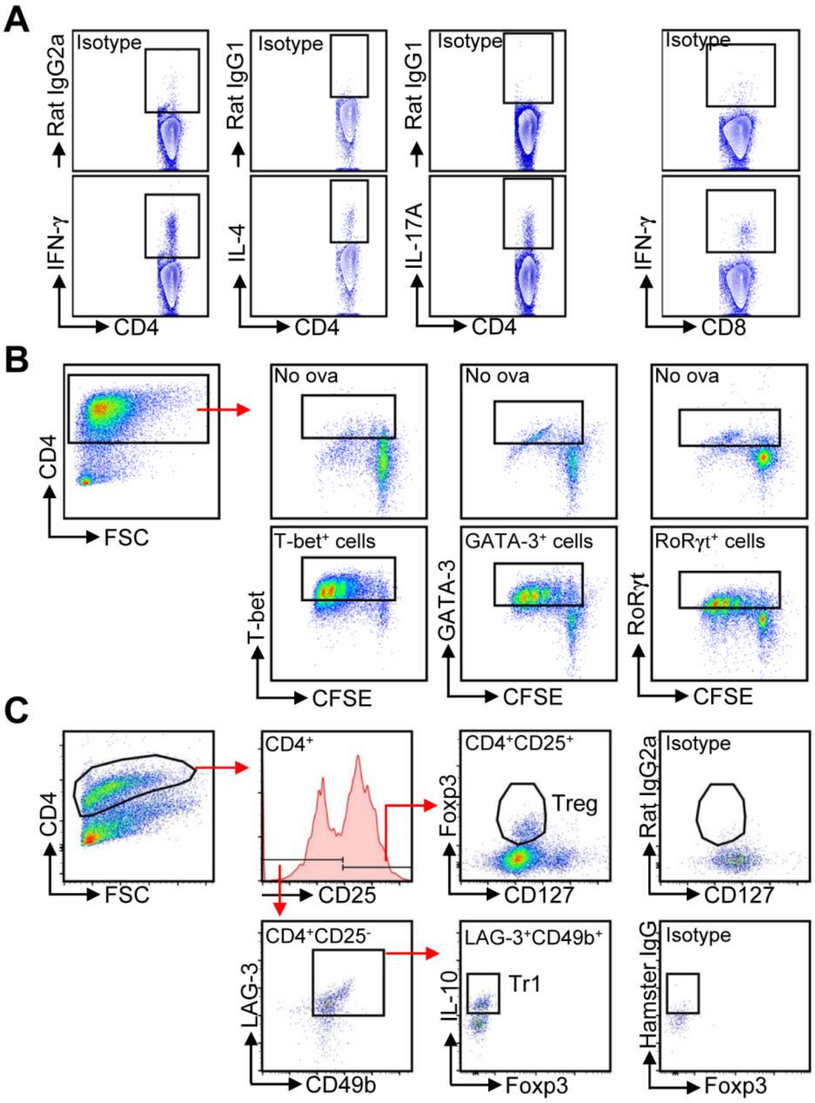

Supplementary Figure S7: Flow cytometry analysis of T cell proliferation and subtypes. To analyze Th1 (IFN-γ$^+$CD4$^+$ or T-bet$^+$CD4$^+$), Th2 (IL-4$^+$CD4$^+$ or GATA-3$^+$CD4$^+$), Th17 (IL-17A$^+$CD4$^+$ or RoRγt-3$^+$CD4$^+$) and activated CD8 (IFN-γ$^+$CD8$^+$) cell populations, inclusion gates (lymphocytes) were drawn based on characteristic FSC and SSC patterns. CD4$^+$ and CD8$^+$ T cells were analyzed by gating on cells stained with anti-CD4 and anti-CD8 Abs. **A.** Gating strategy to identify intracellular cytokine production patterns in CD4$^+$ T cells (IFN-γ$^+$CD4$^+$, IL-4$^+$CD4$^+$ and IL-17A$^+$CD4$^+$) and CD8$^+$ T cells (IFN-γ$^+$CD8$^+$). **B.** CFSE-labeled CD4$^+$ T cells were evaluated for the expression of T-bet, GATA-3 and RoRγt. **C.** Sample gating strategy for CD4$^+$CD127$^-$CD25$^+$Foxp3$^+$ Tregs and CD3$^+$CD4$^+$LAG-3$^+$CD49b$^+$CD25$^-$Foxp3$^-$IL-10$^+$ Tr1 sub-populations within the analyzed CD4$^+$ T cells. All positive cells were analyzed in relation to OT-I and OT-II T cells treated with the IgG control or no OVA control.
